# Media pressure and the process of Westernization in the context of body self-assessment among young heterosexual and gay Polish men

**DOI:** 10.1371/journal.pone.0272907

**Published:** 2022-08-22

**Authors:** Bernadetta Izydorczyk, Małgorzata Lipowska, Sebastian Lizińczyk, Mariusz Lipowski, Jakub Wojtas

**Affiliations:** 1 Institute of Psychology, Jagiellonian University, Krakow, Poland; 2 Institute of Psychology, University of Gdansk, Gdansk, Poland; 3 Institute of Applied Psychology, Jagiellonian University, Krakow, Poland; 4 Department of Psychology, Gdansk University of Physical Education and Sport, Gdansk, Poland; Polytechnic Institute of Coimbra: Instituto Politecnico de Coimbra, PORTUGAL

## Abstract

Mass media and social networks portray a unified image of the perfect male body. The intensity and universality of this influence is an important element of the process of Westernization, especially in traditional cultures such as that of Poland. The main aim of the present study was to investigate the differences between Polish gay and heterosexual men in terms of the role played by self-esteem and the level of internalization of sociocultural standards of body appearance as predictors of the development of their body images. The research study was conducted by reference to 19- to 29-year-old Polish heterosexual (*n* = 287) and gay (*n* = 97) men. The variables were measured using Polish versions of the Sociocultural Attitudes towards Appearance Scale-3, the Self-Esteem Scale, and the Multidimensional Body–Self Relations Questionnaire. Statistical analyses identified several variables as the main predictors of body image in both heterosexual and gay young men: self-esteem, information-seeking, perceived pressure and the internalization of sociocultural standards regarding an athletic body image drawn from mass media. The only significant difference between the two groups was the fact that self-esteem, perceived pressure and the internalization of sociocultural standards from mass media did not play a predictive role with respect to Appearance Orientation among the group of gay men.

## Introduction

Mass media are certainly not the only factor explaining the pattern of body image assessment among both women and men, but they do play an important role in this context [1–5]. Recognizing the important role played by existing models of the ideal body image, as indicated by mass media, it should be noted that a variety of factors explain body image. Among the factors that have been identified as explaining body image, apart from the sociocultural influence of the mass media, many studies have indicated the importance of neural, evolutionary and morphological factors in this context [6–11]. In the assessment of the male body and its attractiveness, according to some studies, women assess stronger men as more attractive than weaker men [11], but other studies have indicated a relationship between the male and hairy somatotype and the assessment of the sexual attractiveness of the male body by women (Masculine Somatotype and Hirsuteness) in terms of the determinants of sexual attractiveness to women. Some fMRI studies have indicated that the configuration of the female body is a significant stimulus for men and activates areas of the male brain that are related to reward processing and appetitive behaviour [6]. Other studies of both men and women have indicated differences between genders in terms of the morphological predictors of sexual behaviour and the importance of waist-to-hip ratio (WHR) in assessing a person’s health and sexual attractiveness [7, 8, 12]. Taking into account these findings and the purpose of the research carried out by the present authors (i.e., to investigate the sociocultural predictors of body image and attractiveness in heterosexual and homosexual men), this article focused in particular on assessing the impact of media mass standards on not only the internalization of body image but also experiences of various aspects of one’s own body among heterosexual and homosexual men.

According to observations of contemporary men and women, the intensity and universality of this influence are important elements of the Westernization process [6–8, 13–15]. The objectification (sexualization) of the female body and, increasingly that of the male body as well is an essential part of this process [16–20]. Such objectification becomes manifest in terms of the excessive pursuit of (frequently unrealistic) sociocultural standards of bodily attractiveness, thus promoting negative self-esteem in both men and women [18–22]. The Western model of the ideal male body is directly related to the classic male stereotype, which is characterized by the promotion of excessive body muscularity, physical fitness, masculinity and domination [23, 24],and becomes manifest in physical characteristics such as muscularity, leanness, and fitness [24]. Despite significant uniformity across different ideals of the male appearance, some cross-cultural differences can be seen in this context, for example, in terms of the desire for muscularity or leanness [12, 25]. Moreover, intercultural differences also emerge in terms of the interconnections among the influence of mass media, the pursuit of the ideal body image and physical activity, and patterns of self-esteem, age and lifestyle [26]. These factors show, inter alia, that adolescents, as compared to adult men, exhibit greater concerns regarding their appearance and fears related to self-esteem [27, 28].

### Polish male stereotypes

Poland, despite its location in the very centre of Europe, is among the countries currently undergoing processes of industrialization, modernization, and Westernization following the Communist period, i.e., after 1989 [29]. Despite the cultural changes that occurred after Poland joined the European Union in 2004, Poland remains a traditional country in regard to gender and gender roles [30, 31]. The traditional Polish stereotype for men is mainly associated with their gender role [32–34] rather than precise criteria for physical attractiveness [35, 36]. The stereotypical role of a Polish man is very masculine and does not allow for any feminine behaviours—traditional ideas of masculinity and femininity remain generally favoured [34]. This stereotype can be seen even in the Polish language, according to which the adjective “męski,” which is formed from the noun “man” (mężczyzna), has several meanings; in addition to meaning “characteristic of a man,” it is also a synonym of the word “brave”, while the term “masculinity” (męskość) can refer to courage, a penis, or sexual potency [37]. The stereotypical appearance of a Polish man emphasizes strength and sexual potency—unambiguous features of sexual dimorphism [35, 36]. Elements of appearance such as leanness and fitness—which are characteristic of the Western male body ideal [24]—are not expected of a stereotypical Polish man.

### Self-esteem and the internalization of sociocultural standards regarding body image

The tripartite influence model of body image [38–40] and sociocultural objectification theory [16] can be used to explain the theoretical basis of the process of Westernization and the significance of sociocultural predictors of body image in that context.

The tripartite influence model claims that one’s levels of satisfaction or dissatisfaction with one’s appearance depend on three primary sociocultural variables: peers, parents, and the media. These factors can exert a direct influence by disseminating a uniform message regarding the perfect look or through judgments of people’s appearances; they can also have an indirect influence via internalization of the socially ideal body type and comparisons based on appearance [38, 40, 41].

Dissatisfaction with the appearance of one’s body is mediated by one’s internalization of the Western world’s ideal of the male body [40, 42]; such dissatisfaction is all the stronger because the ideal of beauty is characterized by objectification of the body [39, 43, 44]. Objectification theory, proposed by Fredrickson and Roberts [16], was initially posited as pertaining only to women, emphasizing the sociocultural contexts in which women are presented as sex objects. Many girls and women perceive their bodies from the perspective of an outsider—as objects that are valued for their physical appearance [45]. However, it has been increasingly noted that the process of Westernization, by creating a unified ideal male appearance that is muscular and lean, is associated with men experiencing objectification of their bodies as well and thus to develop dissatisfaction with their appearance [43, 46–49]. Taken together, the sociocultural theory of objectification and the changes in body ideals associated with the process of Westernization emphasize the importance of psychological factors (e.g., self-esteem) and, furthermore, implicate sociocultural factors in the development of body image among young heterosexual and gay men [46, 47, 49–57].

Moreover, for men, levels of muscularity reflect overall fitness, including sexual fitness, which constitutes a key elements in men’s conformity to masculine norms in certain cultures [58, 59].

In recent years, increased attention has been given to the role played by men’s sexual orientation in the internalization of masculine norms in terms of both gender roles [60–62] and appearance [46, 48, 57, 63–67].

Studies examining the roles played by self-esteem and the endorsement of sociocultural standards of appearance in mass media as predictors of body image in young individuals have increasingly included measures of sexual orientation for both men and women [17, 57, 68–70].

The recent literature concerning the relationship between self-esteem and sociocultural standards in mass media has reported diverse and inconclusive results [56, 65, 71–75]. The most commonly studied aspects of bodily self-esteem are the drives for muscularity and leanness [17, 46, 57, 74, 76, 77]. According to most studies, gay men are more likely to report dissatisfaction with their muscularity than are heterosexual men [48, 57, 63, 64, 67, 78].

In a systematic review, He, Sun [49] analysed 75 studies whose results indicated that men from sexual minorities exhibit higher levels of body dissatisfaction than heterosexual men. Based on a meta-analysis of 27 studies comparing the body images of individuals of different sexual orientations, including both heterosexual and gay men, Morrison, Morrison [47] confirmed the same result: gay men are more dissatisfied with their bodies than are heterosexual men.

The differences between lesbian women (1448) and heterosexual (1391) women are smaller, with lesbians being more satisfied with their bodies. According to some studies, the relationship between self-esteem and the sociocultural influence of mass media pertains only to gay, bisexual, and heterosexual men [46, 74, 76, 77, 79]. There could be many reasons underlying the inconclusiveness and variability in the results of studies concerning the predictors of body image in young heterosexual and gay men. One such reason could be differences in the methods used to measure body image: surveys [17, 76], various scales (e.g., the Body Esteem Scale; [35, 80]), or, most commonly, the measurement of only single variables describing body satisfaction and the pursuit of muscularity in men of different sexual orientations [57]. When measuring sociocultural standards regarding appearance, some researchers have used the Sociocultural Attitudes towards Appearance Questionnaire (SATAQ; [38, 57, 81, 82]), while to measure overall self-esteem, other researchers have used the Self-Esteem Scale (SES [83]). Few studies have emphasized on the multifaceted nature of body image while measuring this variable with a standardized tool. The authors of the current study believe that, in addition to body satisfaction and the internalization of sociocultural standards promoting muscularity and athleticism, it is important to study other factors to do justice to the multifaceted nature of the ways in which men assess their body images, such as satisfaction with one’s body, physical wellbeing, preoccupation with physical fitness, attitudes towards body weight, and health behaviours.

It is possible to find studies that explain the role of mass media messages regarding body image in the development of young men’s self-esteem (which also differentiate between heterosexual and gay men); however, these studies have not simultaneously measured the role played by predictors of the sociocultural influence of mass media and/or self-esteem in the context of experiences of one’s relationship with one’s body.

These variables are defined as categories of the multifaceted construct of one’s relationship with one’s body, and the following aspects are measured: satisfaction with one’s own body and its overall appearance, physical fitness and taking care of one’s looks, levels of concentration with respect to controlling one’s body weight, assessing one’s own health, and the way in which one takes care of one’s health [13, 84].

The authors of this article, in line with the holistic model of health, not only searched for empirically documented indicators of overall body satisfaction (which have been studied frequently) but also referred to indicators describing attitudes towards body health in men (heterosexual and gay). Indicators that allow us to recognize care for the health of the body include assessments of the fear of gaining weight and attitudes towards body weight, self-esteem and care for one’s own physical fitness as well as attitudes towards perceived and experienced signals from the body suggesting the state of health or the symptoms of disease.

### Research objectives, variables, and research questions

The main goal of this study was to investigate differences between gay and heterosexual men in terms of the roles of self-esteem and the level of internalization of sociocultural standards regarding body appearance as predictors in the development of their body images. Taking into account the tripartite influence model and the sociocultural mechanisms underlying the influence of body image that it describes [38], the authors identified two independent variables—self-esteem and sociocultural body image and appearance standards in mass media—and one dependent variable, body image. Sociocultural standards regarding body image and appearance in the mass media were defined, in accordance with the literature, as a variable featuring a four-factor structure that describes the individual’s level of internalization of the sociocultural standards regarding body image and physical appearance. This variable also describes the frequency with which the individual consciously seeks information concerning body image and appearance. The first component of this independent variable is pressure and internalization, which describe the individual’s levels of perceived pressure and internalization of sociocultural body appearance standards promoted by mass media (e.g., TV, radio, magazines, newspapers, and advertisements). These standards, after they are internalized, determine one’s attitude towards the appearance of one’s body. The second component of this variable pertains to seeking information regarding one’s body appearance and internalizing that information. The third component is the internalization of sociocultural standards promoting athleticism (muscularity). The fourth component of the discussed independent variable is the individual’s consciously declared seeking of information regarding body image and appearance in mass media. The other independent variable, self-esteem, describes an individual’s positive/negative attitude towards himself.

The dependent (explained) variable is body image; the authors referred to the cognitive model of body image [38] to define body image as a psychological structure described by the factors included in the Multidimensional Body–Self Relations Questionnaire [85].

The following hypotheses were thus formulated:

1. There are differences between gay and heterosexual men in terms of their self-esteem (Self-Esteem Scale—SES), level of sociocultural internalization of body appearance (Sociocultural Attitudes towards Appearance Questionnaire–SATAQ 3) and body image standards (Multidimensional Body–Self Relations Questionnaire—MBSRQ);
2. Self-esteem is a predictor of the body image of gay and heterosexual men;
3. The internalization of sociocultural standards of body appearance drawn from mass media is a predictor of the body image of gay and heterosexual men;
4. Heterosexual men differ from gay men in terms of the predictors of specific aspects of their body image.

## Methods

### Participants

White Polish men between the ages of 19 and 29 were recruited to participate in this study (*N* = 384; 287 heterosexual men and 97 homosexual men) with a mean age of 22.86 (SD = 3.03). The age of the participants was chosen based on the concept of emerging adulthood [86], which refers to the period between adolescence and adulthood (18–29 years of age), as this period plays a significant role in psychosocial development—in particular, in the development of the relationship between self-esteem and satisfaction with one’s bodily appearance. The sample size was determined on the basis of the estimated number of gay men compared to the entire population of Polish men (source: https://portalstatystyczny.pl/liczba-osob-lgbt-w-europie-i-w-polsce/).

The following inclusion criteria were used: declared heterosexual or homosexual sexual orientation; having a normal BMI for one’s age (18.6–24.9; *M*_BMI_ = 23.7; SD = 4.52); no presence or history of eating disorders (i.e., no medical diagnosis of anorexia, bulimia, or compulsive overeating); no bodily disfigurement (e.g., no visible physical disabilities); not undergoing treatment for mental disorders (psychosis, depression, or personality disorders associated with various forms of self-harm, dysmorphia, etc.); and no history of suicide attempts.

One criterion for inclusion in the sample was normal BMI; men with a BMI indicating obesity or a severely overweight condition were not qualified for participation because it was assumed that people with eating disorders and obesity due to the mechanisms of the disease may have a distorted (abnormal) body image. The authors asked each subject about their body weight and height; the actual weight and height of the subjects were not verified directly. Furthermore, bisexual individuals were excluded from the study to allow for uniform subject groups to be constructed in line with the goals of the study.

### Procedure

This research was part of a broader research project pertaining to “Psychological and sociocultural predictors of the development of body image in the Polish population,” which has been ongoing at the Institute of Applied Psychology of Jagiellonian University in Kraków since 2018”. The present research was conducted from December 2019 to March 2020. The study was conducted online and disseminated via Facebook and through LGBT groups. Researchers informed the respondents of the aim of the study and that participation in the study was voluntary and anonymous. The participants responded voluntarily to the invitation posted on internet forums and Facebook. Participants were not paid for the research. The survey was preceded by instructions and a GDPR clause. The data used in this study were part of a larger survey, and the questionnaires on which this study is based took approximately 10 minutes to complete.

The research was conducted in accordance with national and international regulations and guidelines. Written informed consent was obtained from all participants. All research procedures were conducted in line with the 1964 Declaration of Helsinki as well as further amendments concerning studies with human participants. The protocol was also approved by the Research Ethics Committee of Jagiellonian University in Kraków, Poland

### Measures

The Polish versions of the following questionnaires were used in this research: the Sociocultural Attitudes towards Appearance Scale (SATAQ-3 [87], the Self-Esteem Scale (SES [83]), and the Multidimensional Body–Self Relations Questionnaire (MBSRQ [85, 88]).

The ***Sociocultural Attitudes towards Appearance Questionnaire 3*** developed by Thompson et al. [87], specifically its Polish version [82], was used to measure the components of the independent variable pertaining to sociocultural standards. The Polish version of the SATAQ-3 consists of four scales: *Internalization–Pressure* (a 12-item scale that assesses the individual’s level of internalization of sociocultural standards; this scale contains elements that quantify the intensity of internalization of pressure due to sociocultural body image and physical appearance norms promoted by mass media, and the range of possible scores is 12–60); *Internalization–Information-Seeking* (a scale consisting of six items assessing the level of internalization of sociocultural body image norms due to intentionally seeking information from mass media; the range of possible -scores is 6–30); *Internalization—Athlete* (a 4-item scale intended to assess the internalization of athletic body standards; the range of possible scores is 4–20); and *Information* (a 6-item scale that assesses the frequency with which the individual seeks information regarding standards of body image and appearance; the range of possible scores is 6–30). The SATAQ 3 questionnaire was completed by each subject, who reported their answers on a 5-point Likert-type scale. The Cronbach’s alpha coefficients for the Polish adaptation scales were as follows: *Internalization—General* = 0.93, (Polish version = 0.91), *Internalization—Athlete* = 0.80 (Polish version = 0.96), *Pressure* = 0.92 (Polish version = 0.78), and *Information* = 0.96 (Polish version = 0.89).

The ***Self-Esteem Scale*** (SES) developed by Morris Rosenberg [83], adapted to Polish by Łaguna, Lachowicz-Tabaczek [89], was used to measure self-esteem. The SES scale is composed of 10 items to which participants respond on a 4-level scale, where 1 indicates “I strongly agree” and 4 indicates “I strongly disagree.” Scores for 5 out of 10 items are inverted, and the range of possible scores is 10–40. The scale is characterized by high scale score reliability. Cronbach’s alpha for the scale varies between 0.81 and 0.83 depending on the age group under study. The test–retest reliability (one-year time interval) is 0.5. For the Polish group of respondents, α = 0.879.

The ***Multidimensional Body–Self Relations Questionnaire*** (MBSRQ) developed by Thomas Cash (2000, 2015), adapted to Polish by Brytek-Matera and Rogoza [90], was also used. The MBSRQ consists of 69 questions grouped into 10 subscales, which are clustered into four areas: *Appearance****—****Appearance Evaluation* (AE), *Appearance Orientation* (AO), and *Body Area Satisfaction* (BAS); *Fitness****—****Fitness Evaluation* (FE) and *Fitness Orientation* (FO); *Health****—****Health Evaluation* (HE) and *Health Orientation* (HO); *Illness Orientation* (IO); and *Body Weight****—****Overweight Preoccupation* (OP) and *Self-Classified Weight* (SCW). Participants evaluated each item of the questionnaire by reporting their answers on a five-point Likert-like scale ranging from 1 (“definitely disagree”) to 5 (“definitely agree”). The indicators are slightly different for some items: 1 (never), 2 (rarely), 3 (sometimes), 4 (often), and 5 (very often). Furthermore, some items are reverse-coded. The average score for each scale is estimated to measure the respondent’s self-assessment of their body image using the MBSRQ. The range of possible scores is 1–5. The Polish adaptation of this questionnaire was characterized by Cronbach’s α reliability coefficients ranging from 0.53 to 0.83.

Demographic data concerning age, height, and weight were collected. Body mass index (BMI) was calculated based on participants’ physical characteristics. Moreover, participants indicated their sexual orientation—gay or heterosexual—and reported whether they were sexually active. Only individuals who were already sexually active with either a woman or a man were qualified to participate in the study. The participants were also asked whether they had sexual relations with only men, only women, or both men and women to allow us to exclude bisexual individuals. Participants were recruited to the group of heterosexual men if they indicated that they had had sexual intercourse with a woman at least once and subsequently continued their sexual activity exclusively with women. Participants were recruited to the group of gay men if they indicated that they had had at least one sexual encounter with a man and that they subsequently continued their sexual activity exclusively with men.

### Statistical analysis

For the analysis, a research model was adopted according to which the independent variable was sexual orientation and the independent variables were body image, sociocultural attitudes towards appearance and self-esteem. Statistical analyses were performed using SPSS v. 24 software for Windows. First, in line with the research aims and questions, the intensities of all the variables included in the research model were measured. The Kolmogorov–Smirnov test showed that the distribution of the studied variables did not meet the conditions of normal distribution. Mean values and standard deviations were calculated for all variables. Subsequently, the data for gay and heterosexual men were standardized and compared, and the research model of hypothetical relationships between the variables was analysed using regression.

## Results

The final study group consisted of 384 men, including 287 men who declared a heterosexual orientation and 97 gay men. The selection of respondents was purposeful to ensure that the groups of heterosexual and gay men did not differ in terms of age or BMI.

### Sociocultural and psychological characteristics of the study group—differences between groups

The variance of both groups differed significantly. Statistical analyses of group differences in sociocultural and psychological variables were performed using tests for unequal variances (Table 1), also including effect size, which were calculated using Glass’s rank two-series correlation coefficient. Subsequently, a regression model was used to identify predictors for the body image variable among the groups of gay and heterosexual men.

**Table 1:** Comparative analysis between groups of gay men (n = 97) and heterosexual men (n = 287) in terms of the variables included in the research model.

*Feature*		*Gay Men*		*Heterosexual Men*		*Differences*			
		*M*	*SD*	*M*	*SD*	*T*	*df*	*p*	Effect size
					*(95% CI)*				
	Age	23.13	3.34	22.6	2.73	0,11	110,1	0.780	0.01
					(-0,7:0,12)				
	BMI	23.79	6.72	23.68	3.50	0,16	114,1	0,875	0.01
					(-1,30:1,52)				
**MBRSQ**	**Appearance Evaluation**	**3.09**	**1.00**	**3.49**	**0.83**	**-3,53**	**142,8**	**0,001**	**– 0.22**
					(-0,62:-0,18)				
	**Appearance Orientation**	**3.42**	**0.69**	**3.19**	**0.70**	**2,85**	**165,6**	**0,005**	**0.18**
					(0,07:0,39)				
	**Fitness Evaluation**	**2.65**	**0.98**	**3.53**	**0.96**	**-7,67**	**162,0**	**0,001**	**– 0.48**
					(-1,10:-0,65)				
	**Fitness Orientation**	**2.78**	**0.83**	**3.36**	**0.88**	**-5,88**	**173,8**	**0,001**	**– 0.36**
					(-0,78:-0,39)				
	**Health Evaluation**	**3.53**	**0.83**	**3.71**	**0.79**	**-1,82**	**158,8**	**0,070**	**– 0.14**
					(-0,36:0,01)				
	Health Orientation	2.97	0.61	2.95	0.61	0,21	165,3	0,837	0.00
					(-0,13:0,16)				
	Illness Orientation	3.18	0.79	3.06	0.85	1,29	175,4	0,199	0.07
					(-0,07:0,31)				
	**Body Area Satisfaction**	**3.11**	**0.83**	**3.49**	**0.67**	**-4,07**	**140,3**	**0,001**	**– 0.28**
					(-0,56:-0,19)				
	**Overweight Preoccupation**	**2.59**	**0.99**	**2.09**	**0.82**	**4,51**	**143,1**	**0,001**	**0.30**
					(0,28:0,72)				
	Self–Classified Weight	3.09	0.74	2.95	0.71	1,18	160,8	0,238	0.04
					(-0,07:0,27)				
**Self–Esteem Scale**		**26.89**	**4.34**	**29.26**	**4.48**	**-3,03**	**138,4**	**0,003**	**0,01**
					(-3,92:-0,83)				
**SATAQ 3**	**Internalization Pressures**	**29.10**	**5.54**	**23.02**	**5.80**	**-2,46**	**147,6**	**0,015**	**0.29**
					(3,26:8,92)				
	**Internalization Information Seeking**	**15.06**	**1.00**	**13.56**	**0.83**	**4,25**	**145,5**	**0,001**	**0.05**
					(0,20:2,80)				
	Internalization–Athlete	11.59	5.34	11.32	5.01	0,52	170,2	0,604	0.17
					(-0,75:1,28)				
	**Information**	**11.16**	**0.69**	**9.83**	**0.70**	**2,28**	**172,5**	**0,024**	**0.15**
					(0,11:2,55)				

The effect size of sexual orientation on the explanatory variables indicates whether the influence of the intergroup differences is significant or weak and whether this influence is due to sexual orientation. The following variables were found to be significantly influenced by sexual orientation: *Appearance Evaluation*, *Fitness Evaluation*, *Fitness Orientation*, *Body Area Satisfaction*, *Overweight Preoccupation*, and *Internalization Pressures*. The level of significance of the influence of sexual orientation on differences in other variables was small. Thus, self-esteem regarding one’s appearance, individual parts of the body, physical colour, care for physical fitness, and the fear of gaining weight were the variables that could be explained by the sexual orientation of the Polish male respondents.

A comparative analysis of the mean values of the variables for the groups of young gay and heterosexual men revealed significant differences in terms of the following components of the body image variable (Table 1):

1. Heterosexual men were significantly more satisfied with their overall appearance (Appearance Evaluation) and the appearance of individual parts of their body (Body Area Satisfaction); they expressed significantly higher assessments of their physical fitness (Fitness Evaluation, Fitness Orientation) than similarly aged gay men.
2. Gay men were significantly more concerned about being overweight (Overweight Preoccupation) and were characterized by a greater fear of obesity; they reported more frequent weight vigilance and the use of diets to lose weight than similarly aged heterosexual men.
3. The groups of heterosexual and gay men did not differ significantly in terms of other components of body image: taking care of one’s appearance and healthy lifestyle, sensitivity to symptoms of illness and focusing on illness, or internalization of athletic body standards. Moreover, no differences were observed between the groups of men in terms of the mean values of their age and BMI, thus confirming the validity of the recruitment procedure (i.e., the recruitment of young men with a BMI that is within the normal range for their age).

A comparative analysis of the mean values of the variables for the groups of young heterosexual and gay men also revealed significant differences in the explanatory variables (internalization of sociocultural standards regarding appearance taken from mass media–SATAQ 3 –and self-esteem; Table 1):

1. Heterosexual men exhibited significantly higher overall self-esteem than gay men of a similar age. In this context, however, the effect was small but significant.
2. Heterosexual men were characterized by significantly higher levels of internalization of sociocultural standards regarding body appearance than similarly aged gay men. These groups did not differ in terms of their levels of internalization of sociocultural standards regarding athleticism drawn from mass media.

Therefore, it can be concluded that despite the significant differences identified, some of the variables (*Appearance Orientation*, *Health Orientation*, *Self-Esteem Scale*, *Internalization Information-Seeking*, *Information*) were characterized by a low effect size, which was the result of the weak effect of sexual orientation on those variables.

### Sociocultural standards in mass media and self-esteem as predictors of body image in young heterosexual and gay men

The initial statistical analyses examined the significance of the relationships among the studied variables. These analyses revealed a number of significant correlations among the variables, which allowed for verification of the correlational and regression hypotheses. Forward stepwise regression analysis was used to identify potentially significant predictors.

The regression analyses conducted revealed that self-esteem had a positive influence on 5 of 10 studied sociocultural variables in both groups: Appearance Evaluation, Body Area Satisfaction, Fitness Evaluation, Health Evaluation, and Health Orientation. That is, the higher an individual’s level of self-esteem is, the higher the levels of these variables. Comparison of the values of significant beta coefficients indicates that these were slightly higher for the gay group than for the heterosexual group.

The Internalization Pressures variable significantly influenced 7 of 10 studied sociocultural variables. A positive influence was found for the following variables: Overweight Preoccupation (only for the heterosexual group) and Self-Classified Weight. A negative impact of this variable was found for Appearance Evaluation, Body Area Satisfaction, Fitness Evaluation, Fitness Orientation (only for the heterosexual group), and Health Evaluation.

The Internalization–Information-seeking variable positively influenced only the Health Orientation variable and only for the heterosexual group. No significant influence was observed for the gay group.

Regression analysis revealed that scores on the Internalization-Athlete subscale were positively associated with Fitness Orientation and Health Orientation. A negative influence of this variable was observed with respect to Self-Classified Weight, although this influence was observed only for the gay group.

With respect to the Information variable, a regression analysis indicated a positive influence on Fitness Orientation only for the heterosexual group and a significantly negative influence on Health Evaluation only for the gay group.

## Discussion

### Satisfaction with appearance among Polish gay and heterosexual men

When analysing the self-esteem of the gay and heterosexual participants, the authors found that heterosexual men were characterized by significantly higher levels of overall self-esteem than similarly aged gay men. In contrast, research by Olivardia, Pope [74] showed that heterosexual and gay men do not differ in terms of their overall self-esteem. In the current study, heterosexual men also assessed their health more highly than did their gay peers. No similar measurements of the relationship between the investigated variables among a population of gay and heterosexual men of similar age and BMI could be found in the literature from the past two years. However, a study by Bränström, Hatzenbuehler [91] showed a relationship between young men’s perception of their physical health and their sexual orientation. Similarly, Blashill, Tomassilli [92] surveyed 131 gay and bisexual men and found a significant association between body dissatisfaction and symptoms of depression (i.e., as body dissatisfaction increases, so do symptoms of depression).Yean, Benau [57] also reported that in comparison to heterosexual men, gay men reported significantly more dissatisfaction with their body shape and more symptoms of eating disorders. Their results support the idea that the internalization of sociocultural standards from mass media influences the emergence of eating disorders both directly and indirectly (via various elements of dissatisfaction with one’s body image and low self-esteem).

### Self-esteem and sociocultural predictors of body image in young heterosexual and gay Polish men

The current study also found that self-esteem and internalization of and perceived pressure due to sociocultural standards regarding body image, including standards concerning an athletic body, are the most important predictors of most of the factors that describe body image among young heterosexual and gay Polish men. Only one factor in the dependent variable—Appearance Orientation—was not explained by self-esteem or any factors of the body image variable with respect to the group of gay men. Among the gay group, Health Orientation was not explained by any of the variables according to the model used in this paper. The literature from the past few years contains results that are coherent with ours regarding dissatisfaction with one’s body, satisfaction with one’s body, and internalization of sociocultural standards and/or overall self-esteem among both men and women [18–22]. The significant correlations among the internalization of sociocultural standards, self-esteem, and assessment of one’s body among both men and women should be emphasized, which is in line with the theory of body objectification and the intensification of the process of Westernization in the general population [20]. As shown by the current study, in gay men, higher fat anxiety and dissatisfaction with one’s body can foster excessive pursuit of unrealistic (but persistent) internalized sociocultural attractive body standards, thereby leading to overall negative self-esteem for both men and women [18–22]. Moreover, research concerning young Iranians [26] has shown that media pressure not only affects body esteem but may also be associated with an increased risk of body dysmorphic disorders. The significant sociocultural influences on the development of body image in the population explain and support the tripartite influence model developed by Thompson, Heinberg [38], which has been the focus of many studies. This model has been examined numerous times with respect to populations of heterosexual women [93–95], but research is decidedly rarer with respect to populations of homosexual women [96] and even less common regarding populations of both heterosexual and gay men [97]. However, very little research has measured the influence of sociocultural standards drawn from mass media in the context of multidimensional constructs of body image (as was done in the current study).

Yean, Benau [57] measured the relationship between self-esteem and body image by reference to a sample of 693 individuals (246 men: 130 heterosexual, 101 gay, 15 bisexual; 447 women: 361 heterosexual, 38 lesbian, 48 bisexual). The respondents were young adults with a mean age of 21.23 years—similar to the age of the participants in the current study. Among heterosexual men, internalization predicted 19%, 21%, 22%, and 12% of variance in overall dissatisfaction with one’s body, pursuit of leanness, pursuit of muscularity, and self-esteem, respectively. Among gay/bisexual men, internalization of sociocultural standards predicted 25%, 21%, 18%, and 10% of variance in overall dissatisfaction with one’s body, pursuit of leanness, pursuit of muscularity, and self-esteem, respectively. The results reported by Yean, Benau [57] and Dakanalis and Riva [46] suggest a relationship between self-esteem and body dissatisfaction (i.e., specifically with the upper body: chest, shoulders, and arms) and the pursuit of muscularity in young men. Other studies referencing samples of gay, bisexual, and heterosexual men [74, 77] have reported that pursuit of a lean body by the studied men correlated negatively with their self-esteem and pursuit of an athletic build. Hoffmann and Warschburger [68] also reported the existence of a similarly strong relationship between the internalization of Western body image standards, which are spread by the process of Westernization, and the behaviours of a population of teenagers and young adults regarding their bodies. In their research, Hoffmann and Warschburger [68] prospectively studied two paths: the “weight/shape pathway,” which combines internalization of the ideal of a thin body shape, preoccupation with weight/shape, and restricting food, and the “muscularity pathway,” which combines internalization of the ideal of an athletic body, preoccupation with muscularity, and behaviours oriented towards increasing one’s muscularity. Hoffmann and Warschburger [68] reported that body weight and the associated body appearance are important with respect to predicting restrictive eating behaviours, while pursuit of muscularity predicted pursuit of an athletic build in both genders.

## Conclusions

Table 2 presents a summary of the similarities and differences between heterosexual and gay men in terms of the studied predictors of body image according to the research model.

**Table 2:** Summary of significant predictors of the body image variable in the studied groups.

Explained Variables Body Image	Regression models Explanatory variables Sociocultural standards in mass media and self-esteem	
	Heterosexual men	Gay men
Appearance Evaluation	Self-esteem β = 0.44*** Internalization–pressure β = –.21*** R^*2*^ = .22, *F*(3, 283) = 27.39, *p* < .001	Self-esteem β = 0.50*** Internalization–pressure β = –.43*** R^*2*^ = .35, *F*(3, 93) = 17.93, *p* < .001
Appearance Orientation	Internalization–pressure β = .15* Internalization–athlete β = .22*** Internalization–information-seeking β = .15* R^*2*^ = .15, *F*(3, 283) = 18.21, *p* < .001	-
Fitness Evaluation	Self-esteem β = .22***. Internalization–athlete β = .24*** Internalization–pressure β = –.34** Information β = .20* R^*2*^ = .09, *F*(4, 282) = 8.02, *p* < .001	Self-esteem β = 0.34*** Internalization–pressure β = –.30** Internalization–athlete β = .25* R^*2*^ = .15, *F*(3, 93) = 6.52, *p* < .001
Fitness Orientation	Internalization–athlete β = .52*** Internalization–pressure β = –.40*** Information β = .22* Self-esteem β = .11* R^*2*^ = .20, *F*(4, 282) = 18.31, *p* < .001	Internalization–athlete β = .55*** Self-esteem β = .26** Internalization–pressure β = –.35*** R^*2*^ = .23, *F*(4, 92) = 8.05, *p* < .001
Health Evaluation	Self-esteem β = .28*** Internalization–information-seeking β = –.17** Internalization–athlete β = .22*** Internalization–pressure β = .21** R^*2*^ = .18, *F*(4, 282) = 16.95, *p* < .001	Self-esteem β = .44*** Internalization–pressure β = –.52*** Information β = .31* R^*2*^ = .27, *F*(4, 92) = 10.07, *p* < .001
Health Orientation	Internalization–athlete β = .35*** Self-esteem β = .24*** Internalization–Information-seeking β = .17** Internalization–pressure β = –.28* Information β = .22* R^*2*^ = .16, *F*(5, 281) = 11.78, *p* < .001	Self-esteem β = .31*** Internalization–athlete β = .36** R^*2*^ = .14, *F*(3, 93) = 6.06, *p* < 0.001
Illness Orientation	-	-
Body Area Satisfaction	Self-esteem β = .38*** Internalization–pressure β = –.27*** Information β = .19* R^*2*^ = .20, *F*(4, 282) = 18.91, *p* < .001	Self-esteem β = .58*** Internalization–pressure β = –.27*** R^*2*^ = .43, *F*(2, 94) = 37.37, *p* < .001
Overweight Preoccupation	Internalization–pressure β = .39*** R^*2*^ = .17, *F*(2, 284) = 29.31, *p* < .001	Self-esteem β = –.22*** R^*2*^ = .15, *F*(3, 93) = 6.69, *p* < .001
Self-Classified Weight	Internalization–pressure β = .35** Information β = –.24* R^*2*^ = .03, *F*(3, 283) = 4.16, *p* < .01	Internalization–pressure β = .46*** Internalization–athlete β = –.32** R^*2*^ = .11, *F*(2, 94) = 6.86, *p* < .01

Statistical analyses indicate that overall self-esteem and internalization–information-seeking as well as perceived pressure and the internalization of sociocultural standards regarding body image from mass media, especially the internalization of athletic body standards, are the main predictors of body image in both heterosexual and gay young men. The only statistically significant difference between these groups is the fact that self-esteem, perceived pressure and the internalization of sociocultural standards from mass media do not play a predictive role with respect to Appearance Orientation among gay men.

## Limitations

The current study exhibits certain limitations. First, the sample included in the study might not have been representative of gay men in Poland as they were difficult to contact. Future studies should attempt to recruit more participants from this population. Second, this research was largely cross-sectional, and while some correlational–regression hypotheses were proposed, it was difficult to draw clear-cut conclusions. However, the study employed self-report questionnaires, which are characterized by a high degree of statistical reliability, thus supporting the validity of the results. The groups of gay and heterosexual participants included in the study did not differ significantly in terms of age or level of education; thus, it can be said that the results are representative of the relationship between the variables only in the case of young men. To obtain fully representative data, more diverse groups of subjects (in terms of age, level of education, etc.) should be studied. Moreover, we included only individuals who were biologically male. However, extending this research to include questions related to psychological gender would inevitably result in a transition to a different topic. Despite these limitations, the study makes an important contribution to the scientific measurement of the roles played by self-esteem and sociocultural standards in shaping the body images of young heterosexual and gay men.
